# Combination of Drugs and Cell Transplantation: More Beneficial Stem Cell-Based Regenerative Therapies Targeting Neurological Disorders

**DOI:** 10.3390/ijms22169047

**Published:** 2021-08-22

**Authors:** Kaneyasu Nishimura, Kazuyuki Takata

**Affiliations:** Division of Integrated Pharmaceutical Science, Kyoto Pharmaceutical University, Kyoto 607-8414, Japan; kaz@mb.kyoto-phu.ac.jp

**Keywords:** cell transplantation therapy, drugs, combined therapy, neurodegenerative diseases, pluripotent stem cells, multipotent stem cells

## Abstract

Cell transplantation therapy using pluripotent/multipotent stem cells has gained attention as a novel therapeutic strategy for treating neurodegenerative diseases, including Parkinson’s disease, Alzheimer’s disease, Huntington’s disease, ischemic stroke, and spinal cord injury. To fully realize the potential of cell transplantation therapy, new therapeutic options that increase cell engraftments must be developed, either through modifications to the grafted cells themselves or through changes in the microenvironment surrounding the grafted region. Together these developments could potentially restore lost neuronal function by better supporting grafted cells. In addition, drug administration can improve the outcome of cell transplantation therapy through better accessibility and delivery to the target region following cell transplantation. Here we introduce examples of drug repurposing approaches for more successful transplantation therapies based on preclinical experiments with clinically approved drugs. Drug repurposing is an advantageous drug development strategy because drugs that have already been clinically approved can be repurposed to treat other diseases faster and at lower cost. Therefore, drug repurposing is a reasonable approach to enhance the outcomes of cell transplantation therapies for neurological diseases. Ideal repurposing candidates would result in more efficient cell transplantation therapies and provide a new and beneficial therapeutic combination.

## 1. Introduction

The central nervous system of adult humans is not innately repaired following the loss of neuronal cells due to neurological damage and diseases. Despite ongoing substantial efforts over many decades, successful therapies have not yet been established for several neuronal disorders, including Parkinson’s disease (PD), Alzheimer’s disease (AD), Huntington’s disease (HD), ischemic stroke, and spinal cord injury (SCI). Current therapies include medications, surgical treatment, and rehabilitation, but these interventions can only help to manage the pathological symptoms of diseases and cannot restore lost neuronal function or ultimately provide cures. Therefore, although the currently available therapies may provide some symptomatic relief, existing therapeutic procedures neither slow disease progression nor repair damaged structures.

Recently, cell transplantation therapy has gained substantial attention as a novel therapeutic strategy in the treatment of neurological diseases. Pluripotent stem cells (PSCs), including embryonic stem cells (ESCs) and induced pluripotent stem cells (iPSCs), are promising sources of cell-based regenerative therapies because these cells give rise to defined cell types at necessary quantities and scalabilities [1,2]. Recent advantages in PSC technology include the ability to generate clinical-grade donor cells in the treatment of neurological diseases, including age-related macular degeneration (AMD), PD, HD, and SCI, in which lost neurons can be functionally repaired. Therapeutic benefits of iPSCs extend to autologous transplantation, as they may prevent negative immune responses such as immune rejection following cell grafting [3,4,5,6]. Numerous clinical and preclinical studies have shown that transplantation of dopaminergic (DA) progenitors, medium spiny neurons, and neural precursor cells (NPCs) are promising therapeutic strategies that could aid in the recovery of parkinsonian motor symptoms and motor function in HD and SCI, respectively. Several groups have established robust and scalable protocols to obtain donor cells from human PSCs (hPSCs), and they have demonstrated the efficacy and safety of grafted cells with long-term trials in animal models, including rodents and non-human primates [7,8,9,10,11,12]. These grafted cells can survive and resume their appropriate function in the nervous system by replacement of lost neuronal function.

Whilst cell-based regenerative medicine has substantial promise, significant challenges remain. Donor cells should meet criteria regarding safety, efficacy, stability, reproducibility, and scalability before transplantation into patients. Additionally, protocols surrounding good manufacturing practice-grade production should be developed using quality control tests at a facility dedicated to clinical trials. Cell grafts result in significant improvement in the quality of life for patients and their families. For this purpose, several steps should be taken to determine the efficacy of grafted cells, such as cell survival analyses, neuronal maturation, neurite extension to target cells, formation of synapses with target neurons, and functional integration into the host neural circuitry following cell transplantation [13].

Additionally, safety issues including immune rejection of grafted cells by neuroinflammation and tumorigenicity of the grafted cells following cell transplantation must be avoided to provide safer cell transplantation therapy to patients. To address these issues, some therapeutic options have already been developed, including monitoring inflammations and graft size by positron emission tomography imaging, inhibiting tumorigenicity and overgrowth of grafted cells by γ-ray irradiation, and finally surgically removing grafted cells when risks of cell transplantation outweighed the benefits [9,14,15,16,17].

The CNS has endogenous, but not sufficient, potential to compensate for lost neuronal functions by recruiting endogenous neural progenitors, suggesting that grafted-neural progenitors could also respond to their microenvironment [18,19,20,21]. The next step in improving outcomes after cell transplantation should be indirect modification of the microenvironment of the host-targeted region and grafted neurons. If drugs could be successfully targeted and delivered to grafted neurons, medications could be a powerful method for modifying brain microenvironments and preclude invasive surgical operations.

Together with the discovery of cell transplantation therapies, numerous efforts have also been made to inject or systemically administer certain compounds and soluble factors along with grafted cells. For this strategy, an initial aim should be to screen potential reagents from a group of non-approved drugs or materials that can modify the microenvironment of grafted cells, since it is necessary to establish the proof-of-concept for combination therapy of cell transplantation and drugs, and even the usage of non-approved drugs, as a first step. Next, researchers could focus on unrecognized side effects of already-approved drugs on grafted cells as part of a drug repurposing approach. Drug repurposing is advantageous because effective and safe doses of drugs, as well as routes of administration, have already been established. Furthermore, details regarding their pharmacokinetics and contraindications for combined use are also known. Therefore, drug repurposing may provide a therapeutic opportunity that can treat patients more quickly and at a lower cost than new drug development. A combination of cell transplantation therapy and treatment with approved drugs may synergistically improve the outcome for neurological patients.

Here, we show proof-of-concept for conventional approaches with endogenous growth factors have been established and can improve the efficacy of cell transplantation therapy. Next, we discuss the therapeutic significance of repurposed drugs as a replacement for endogenous growth factors and how this may improve outcomes of cell transplantation therapy. We also highlight examples of preclinical studies using repurposed drugs that improve the efficacy of cell transplantation therapy for neurological diseases. Finally, we discuss current issues and future prospects of combination strategies of cell transplantation therapy and approved, repurposed drugs.

## 2. Translational Approach of Endogenous Growth Factors for Effective Cell Transplantation Therapy

Endogenous neurotrophic factors such as glial cell-derived neurotrophic factor (GDNF), brain-derived neurotrophic factor, and nerve growth factor are secreted from glial cells including astrocytes, oligodendrocytes, and microglia. These factors all play important roles in neural development, maturation, and survival during brain development [22]. Endogenous neurotrophic factors are expected to be able to help support grafted neuronal cells. For instance, GDNF is an endogenous soluble factor that exerts a potent effect on cell survival and the maturation of DA neurons by stimulating the GDNF receptor RET in cell culture systems and in animal models of PD. GDNF was examined to determine whether it could support grafted DA neurons and result in better outcomes following cell transplantation experiments, and results from an animal study indicated that GDNF increased the survival and maturation of grafted DA neurons [23,24,25]. These studies revealed that endogenous factors, which have protective effects both in vivo and in vitro, are translatable to cell transplantation. Although GDNF treatment moved to clinical trials, GDNF has since been withdrawn, as the anti-GDNF antibody was produced in the recipient patients [26]. Recent reports suggested that a novel small molecule, BT13, could bind to RET and activate its downstream signaling cascades [27]. BT13 protects DA neurons from neurotoxin-induced cell death in vitro. Additionally, BT13 activates DA signaling via RET and promotes the release of DA in the striatum in vivo, suggesting that BT13 is a candidate compound for PD therapy possibly applicable for successful cell engraftment and warranting further study [28].

## 3. Drug Repurposing Approach toward Effective Cell Transplantation Therapy

**Figure 1:**
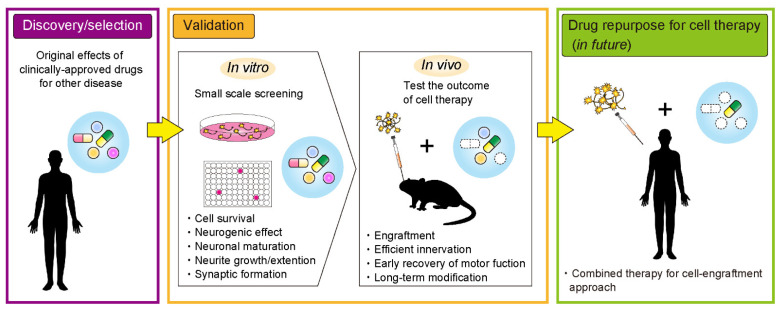
An overview of the drug repurposing process towards more effective cell transplantation therapy for neurological diseases.

In order for more effective neuronal transplantation therapy, cell survival, neuronal maturation, neurite extension, and formation of synapses with host neurons are all necessary. To achieve these, therapeutic options in clinical and preclinical studies have included gene therapy, rehabilitation, and medication [29,30,31,32]. Medication is considered the most simple treatment option with regards to patient burden and avoids invasive surgical operations. Several drug candidates for repurposing could be applied as part of a combination strategy for cell transplantation therapy and pharmacotherapy to improve outcomes of cell transplantation therapy (Figure 1). For a combination strategy to work, drugs must be appropriately delivered to the grafted cells and manipulate the microenvironment of the grafting site. Potential examples of drug repurposing for more successful transplantation therapy are described below.

### 3.1. Immunosuppressants

For decades, immunosuppressant drugs such as cyclosporine A (CsA) and tacrolimus (TAC, also known as FK506) have been widely used to suppress the immune rejection of grafted tissues and cells in cases of allo- and xeno-transplantation. Recent clinical study suggested that immunosuppressant drugs were not needed for transplantation therapy using autologous iPSC-derived neurons [33]. However, it still needed to suppress the immune response in cases of allogenic transplantation using ESC- and allogenic iPSC-derived neurons [34]. CsA suppresses the activation of lymphocytes by blocking interleukin (IL)-2 synthesis through inhibition of the calcineurin pathway [35]. CsA improved cell survival after human fetal DA neurons were grafted into a rat model of PD through a mechanism that blocked the activation of immune cells [36,37,38]. Based on these preclinical studies, CsA was administered to PD patients who received human fetal tissue in order to minimize the risk of immune rejection [39,40,41,42]. Based on this experimental and clinical evidence, CsA is now widely used to improve the survival of grafted PSC-derived neurons in cross-species studies by systemic administration in various neurological disease animal models including PD, SCI, HD, retinal degeneration, and stroke [8,43,44,45,46,47,48,49,50].

TAC binds the FK506 binding protein (FKBP) and blocks IL-2 production through inhibition of the peptidylprolyl cis-trans isomerase activity of FKBP [51]. TAC exerts a highly potent immunosuppressive effect at concentrations 100-fold lower than CsA [52,53]. TAC is also used for increasing the survival of grafted PSC-derived neural progenitors/organoids in cross-species studies in various neurological disease models [9,12,54,55]. Additionally, recent reports have shown that TAC suppressed the infiltration of lymphocytes into the graft area of hiPSC-derived cells in a major histocompatibility complex-matched/mismatched monkey study [4,55]. Therefore, immunosuppressive reagents are the most promising drugs to support the survival of grafted neurons.

### 3.2. Rho-Kinase Inhibitors

The targeted inhibition of Rho-kinase with Y-27632 drastically improved the survival of undifferentiated hPSCs and the proliferative ability of single-cell-passaged cells [29]. The major issue after cell transplantation is cell death as a result of mechanical stress and the loss of cell–cell contacts due to cell dissociation, low levels of trophic factors, and oxidative stress and hypoxia [56,57]. When mouse ESC-derived neurospheres were grafted into a mouse brain together with Y-27632, cell survival significantly improved as a result of fewer apoptotic cells in the graft [58]. Additionally, Y-27632 was also reported to promote neurite growth in rat NPCs [59], human NPC culture [60], and axonal regeneration in a cat model of optic nerve injury [61]. Therefore, Rho-kinase inhibitors are potent therapies that may increase cell survival and neurite extension and ultimately help in the recovery of neuronal function following cell transplantation.

### 3.3. Chondroitinase ABC (ChABC)

Chondroitin sulfate proteoglycans (CSPGs) are widely expressed in the CNS where they are involved in cell adhesion and growth, receptor binding, and cell migration during development [62]. In response to neuronal injury and disease, astrocytes accumulate at the injured region and secrete CSPGs to form a glial scar, which is a biological barrier that inhibits axonal extension. Glial scars result in failures in neurite extension and regeneration during endogenous repair following injury and disease [63]. After cell transplantation therapy, a glial scar forms and surrounds the grafted cells to inhibit neurite growth and regeneration from the grafted cells and disturbs the appropriate neuronal innervation of the grafted neurons with host neurons. ChABC is an enzyme clinically used in patients with lumbar disc herniation that cleaves the sulfated glycosaminoglycan chain of CSPGs and promotes additional axonal regeneration in the scarred region [64]. A preclinical study on SCI treatment demonstrated that ChABC promotes axonal regrowth by modifying the plasticity of myelinated tracts [65,66]. Because cell transplantation is an invasive procedure that evokes local accumulations of astrocytes and microglia, as well as inflammation, glial scars form along the grafted site. One benefit of cell transplantation is the ability of grafted neurons to promote axonal innervation of host neurons following cell transplantation to restore lost neuronal functions. Based on this observation, several groups have examined the ability of ChABC in some types of neuronal cells to increase neurite extension and integration of grafted cells into the host neural circuitry. It was shown that ChABC, when injected into the medial forebrain bundle, cleaved CSPGs along the nigrostriatal pathway and promoted the reinnervation of grafted DA neurons into the striatum in an animal model of PD [67]. In cell transplantation therapy for SCI with either hiPSC-derived NPCs or hiPSC-derived neuroepithelial cells, ChABC promotes tissue repair and cell survival with long-term functional recovery of motor activity [68,69].

### 3.4. Valproic Acid (VPA)

VPA is used clinically for long-term treatment of epilepsy and mood disorders. VPA has been reported to inhibit histone deacetylases, leading to hyperacetylation of histones, in addition to mediating the neuronal differentiation of adult hippocampal neural progenitors [70]. VPA also induced an increase in neuronal gene expression during the differentiation of hESCs into neurons [71]. An in vivo study using an SCI rat model demonstrated that VPA administration enhanced the neurogenic potential of neural stem/precursor cells after SCI and gene expression of neuronal markers around the injured spinal cord. Motor function also recovered as a result of VPA administration following SCI [72]. Taken together, VPA administration is expected to increase the neurogenic potential of neural stem/precursor cells following neuronal injury in addition to promoting functional recovery following cell transplantation for neuronal injury. Previous reports have shown that VPA administration enhanced the restoration of hindlimb function when mouse neural stem cells were transplanted together with VPA in a mouse model of SCI. According to a histological analysis, VPA administration promoted neuronal differentiation rather than glial differentiation [73]. When neonatal Nrl^+^ retinal cells were grafted with VPA into the eyes of rd mice, which have a phosphodiesterase 6b gene mutation and exhibit rapid degeneration of retinal cells, the number of integrated cells into the eye increased following VPA supplementation. Further, an increased number of rhodopsin^+^ cells was also observed in the early treatment period [74]. VPA administration increased the number of NeuN^+^ neurons and tyrosine hydroxylase (TH)^+^ DA neurons in the grafts of mouse iPSC-derived DA progenitors and increased the number of DA neurons transplanted into the striatum of normal mouse [75]. These reports suggest that both systemic administration and local injection of VPA potentiate neuronal differentiation of grafted-neural cells.

### 3.5. Zonisamide

Zonisamide is widely used as an anti-epilepsy and anti-parkinsonian drug. Recent findings have shown that zonisamide has neuroprotective and neuroregenerative effects in cases of neurological injury. Further, recent studies demonstrated that zonisamide rescued the loss of DA neurons following 1-methyl-4-phenyl-1,2,3,6-tetrahydropyridine treatment-induced toxicity in parkinsonian animal models by activating tyrosine hydroxylase activity through the inhibition of monoamine oxidase B [76,77]. An additional report suggested that the inhibition of microglial activation by zonisamide mediates the survival of DA neurons [78]. Electrophysiological analysis revealed that zonisamide protects striatal neurons against rotenone-induced mitochondrial impairment [79]. Zonisamide also exerts a protective effect against oxidative stress-induced cell death of primary motor neurons and enhances neurite elongation and regeneration via an increase in nerve growth factors. In a mouse model in which a sciatic nerve autograft was used, zonisamide improved the sciatic functional index, which served as a marker of hindlimb motor functional recovery after sciatic nerve autograft procedures were performed [80]. Repeated administration of zonisamide enhanced the number of surviving mouse iPSC-derived DA neurons following cell transplantation into the mouse striatum [75]. Long-term daily administration of zonisamide also promoted the survival of grafted hiPSC-derived DA neurons in 6-hydroxydopamine (6-OHDA)-lesioned rat striatum via the activation of SLIT and NTRK like protein 6; these proteins are expressed in the striatum, which was the target region of cell transplantation [81].

### 3.6. 17β-Estradiol (E2)

E2 is a gonadal steroid hormone and an essential female sex hormone. Clinically, E2 and its derivative, estradiol-2-benzoate (E2B), are used to reduce menopausal and age-related symptoms in women. E2 has been reported to exert protective effects against neurotoxic reagents and oxidative stress in cell culture systems [82,83]. Administration of E2 to MPTP-treated parkinsonian rodent models exhibited protective effects against neuronal death by inhibiting oxidative stress and inflammation, and a low dose of E2 administered to rats with SCI resulted in improved motor function by decreasing inflammation, tissue damage, gliosis, and neuronal cell death [84,85,86,87]. Therefore E2, when combined with cells, could be applicable for cell transplantation therapy in SCI. Recently, several studies have reported on the combination therapy of E2 and transplantation of Schwann cells for the treatment of SCI in animal models [88,89,90]. Additionally, it has been reported that systemic and repeated administration of E2B affects the activation of integrin α5, which is selectively expressed in striatal medium spiny neurons innervated by DA neurons in adult female rodents [91]. Integrin α5 can be activated through upregulation of the reelin pathway, and because integrin α5β1 has a high affinity for fibronectin, cell–cell interactions between striatal neurons and hiPSC-derived DA neurons could be facilitated by the activation of integrin α5β1 [92,93]. Systemic administration of E2B to 6-OHDA-lesioned parkinsonian rats promoted synaptic input to the host striatal neurons from the grafted iPSC-derived DA neurons and resulted in earlier recovery of motor function. Additionally, levels of integrin α5 transcripts in the postmortem putamen of PD patients were the same as those of healthy controls. Together these findings suggest that E2B is a drug that can potentially support cell transplantation therapy.

## 4. Future Prospects

In this review, we introduced an overview of drug repurposing approaches to emphasize its potential role in cell transplantation therapies for treatment of neurological diseases. Current drug repurposing approaches have identified candidate drugs through pharmacotherapy for other diseases, coincidences, or bioinformatic approaches from accumulated clinical cases [94,95]. The number of cases of cell transplantation therapy using pluripotent or multipotent stem cells remains low, as it is a developing treatment approach with clinical trials ongoing worldwide [96,97]. Because of these reasons, the number of patients who have received both cell transplantation and pharmacotherapy is not sufficient for identifying new and successful drug(s) for drug repositioning. Therefore, minimal opportunities exist for determining new combinations of conventional drugs to increase the therapeutic benefit of cell transplantation. However, numerous preclinical studies are underway to establish the proof-of-concept that clinically approved drugs can support the outcome of cell transplantation therapy. Recent progress has used clinical-grade donor cells-derived from hPSCs in transplantation experiments using animal models [5,7,10,98]. In the future, more preclinical and clinical cases will contribute to the search for new candidate drugs that support cell transplantation therapy.

On the other hand, issues have been raised that question the efficacy and safety of drug repurposing to support cell transplantation therapy. For instance, some already-approved drugs, especially non-CNS drugs, are unable to cross the blood brain barrier and are difficult to reach the CNS. Additionally, transcellular transporters such as the ATP binding cassette (ABC) transporter expressed in brain endothelial cells limit the efficacy of any drug treatment by controlling penetration and excretion of the drugs [98]. Therefore, the administration route and drug delivery approach must be considered for CNS targeting if repurposed drug(s) are not developed as CNS drugs. The appropriate dose, administration period, and dosing frequency for cell transplantation therapy will also need to be re-optimized for each drug, since current administration conditions are used in the context of other diseases. Another limitation of drug repurposing is that aging is a non-modifiable risk factor for AD, PD, and stroke. If repurposed drugs are not originally for use in elderly individuals, pharmacokinetics and pharmacodynamics of repurposed drugs will need to be considered to determine optimal administration [99,100]. Additionally, unexpected side effects should be expected by combining repurposed drugs with cell transplantation therapy. The safety profiles of repurposed drugs will need to be optimized for use in cell transplantation therapy. The results of current candidates are only suggestions from preclinical studies and more data must be accumulated before clinical trials are conducted with these candidates. Bioinformatic approaches in particular could help develop useful prediction tools to identify additional candidate drugs in the future. Bioinformatic tools can predict and evaluate biological significance, experimental and clinical data, and chemical structure of candidates [95,101].

## 5. Conclusions

In this review, we highlighted several potential candidate drugs that can be used for more effective cell transplantation therapy of neurological diseases. These candidate drugs were selected from preclinical studies that demonstrated the effective combination of cell transplantation therapy with repurposed drugs. More clinical cases should be studied to identify additional repurposed drugs. We expect that a novel combination therapy consisting of cells and drugs would benefit patients as a next-generation approach, as new stem cell-based regenerative therapies are also being developed. Drug repurposing may become a beneficial approach for combination therapy of cell transplantation and pharmacotherapy and may lead to better outcomes of stem cell-based regenerative medicine.
